# MicroRNA Profiling in *Muc2* Knockout Mice of Colitis-Associated Cancer Model Reveals Epigenetic Alterations during Chronic Colitis Malignant Transformation

**DOI:** 10.1371/journal.pone.0099132

**Published:** 2014-06-18

**Authors:** Yonghua Bao, Yongchen Guo, Zexin Li, Wenfeng Fang, Yiqiong Yang, Xuhan Li, Zhuangzhuang Li, Bowen Xiong, Zhiguo Chen, Jianguo Wang, Kang Kang, Deming Gou, Wancai Yang

**Affiliations:** 1 Department of Immunology, Xinxiang Medical University, Xinxiang, China; 2 Department of Laboratory Medicine, Xinxiang Medical University, Xinxiang, China; 3 Department of Surgery, the First Affiliated Hospital of Xinxiang Medical University, Weihui, China; 4 Department of Oncology, Sun Yat-Sen University Cancer Center, *G*uangzhou, China; 5 School of Basic Medical Sciences, Xinxiang Medical University, Xinxiang, China; 6 School of Life Sciences, Shenzhen University, Shenzhen, China; 7 Department of Pathology, University of Illinois at Chicago, Chicago, Illinois, United States of America; Fox Chase Cancer Center, United States of America

## Abstract

Our previous studies have demonstrated that genetic deletion of the *Muc2* gene causes colorectal cancers in mice. The current study further showed that at the early stage (<3 months) the *Muc2* knockout mice spontaneously developed chronic inflammation in colon and rectum, similar pathological features as human colitis; and at the late stage (>3 months) the mice exhibited colorectal cancer, including a unique phenotype of rectal prolapsed (rectal severe inflammation and adenocarcinoma). Thus, the age of 3 months might be the key point of the transition from chronic inflammation to cancer. To determine the mechanisms of the malignant transformation, we conducted miRNA array on the colonic epithelial cells from the 3-month *Muc2* ^−/−^ and ^+/+^ mice. MicroRNA profiling showed differential expression of miRNAs (i.e. lower or higher expression enrichments) in *Muc2* ^−/−^ mice. 15 of them were validated by quantitative PCR. Based on relevance to cytokine and cancer, 4 miRNAs (miR-138, miR-145, miR-146a, and miR-150) were validate and were found significantly downregulated in human colitis and colorectal cancer tissues. The network of the targets of these miRNAs was characterized, and interestedly, miRNA-associated cytokines were significantly increased in *Muc2* ^−/−^mice. This is the first to reveal the importance of aberrant expression of miRNAs in dynamically transformation from chronic colitis to colitis-associated cancer. These findings shed light on revealing the mechanisms of chronic colitis malignant transformation.

## Introduction

Colorectal cancer (CRC) is the third common malignant disease and the second leading causes of cancer-related death [1]. Similar as other malignancies, genetic factors contribute a lot to CRC formation, but, only about 20% of CRC cases can be genetically attributed to familiar history [2]–[4]. In fact, most of sporadic CRC are strongly linked to environmental factors, by which the most often mutations in adenomatous polyposis coli (APC) tumor suppressor gene lead to destruction of Apc/GSK3β/Axin complex and activation of Wnt/β-catenin pathway [2], [5], [6] Aberrant activation of Wnt/β-catenin pathway not only promotes proliferation of intestinal epithelial cells but also induces their arrest as they move towards the end of the crypt and prevent shedding or apoptosis of transformed cells. The canonical “genetic pathway to colorectal cancer” has been well studies. The emerging mechanisms of colorectal cancer formation by environmental factors are associated with chronic inflammation, named colitis-associated colorectal cancer (CAC) [7]–[10] With the changes of diets and life-style in China, CRC incidence rate increases rapidly than any other cancers in recent years [11], and quite amount of CRC cases are linked to chronic inflammatory bowel disease (IBD). It is acceptable that CAC is preceded by clinically detectable IBD [8], [12], such as Crohn’s disease (CD) or ulcerative colitis (UC). Epidemiology and clinical studies have suggested that UC increases CAC risk by up to 18–20%, while CD by up to 8% after 30 years of active disease [13]–[15]. In mouse models, single injection of carcinogen azoxymethane (AOM) leads to multiple colonic tumors, only when coupled with chronic colitis induced by dextran sodium sulfate (DSS), while when inflammation is absent it takes multiple injections of AOM and longer time for tumor formation [16], [17]. These clinical and experimental observations clearly pinpoint CAC as classical inflammation-driven cancer. However, unlike Apc/Wnt/β-catenin pathway, the mechanisms underlying colitis-associated cancer, and particularly, of colitis malignant transformation, are largely unclear, majorly due to lack of an appropriate model for dynamical investigation.

Intestinal epithelia are protected by a layer of mucin secreted by goblet cells against mechanical and chemical injuries, potent causes of inflammation, and the most abundant secreted intestinal mucin is encoded by the *Muc2* gene [18]. Previous studies have shown that decreased number of goblet cells and reduced *Muc2* expression is commonly observed in ulcerative colitis and colorectal carcinoma [19]. The importance of *MUC2* in intestinal homeostasis is reflected by alterations of cell proliferation, migration and apoptosis in the mouse intestine upon genetic deletion of the *Muc2* gene [20], and most importantly, *Muc2*-deficiency mice (*Muc2* ^−/−^ mice) spontaneously develop small and large intestinal, and rectal, tumors [20]. Mechanistic studies have shown that tumorigenesis is associated with activation of chronic inflammation, and is not associated with Wnt/β-catenin signaling [20]. However, the inflammation increased intestinal tumorigenesis in Apc mutant mice by introducing *Muc2* deficiency to the mice [21], and loss of cyclin dependent kinase inhibitor p21WAF1 enhanced intestinal tumor formation in *Muc2* ^−/−^ mice [22], Our current study further showed that *Muc2* deficiency mice spontaneously develop chronic colitis at their early age (<3 moths), whose histopathology was similar to ulcerative colitis in patients. After 3 months, the *Muc2* ^−/−^ mice develop colonic and rectal tumors. Therefore, the age of 3 months might be the key point at which chronic colitis progresses to colorectal cancer, i.e. colitis malignant transformation, and the *Muc2* mice could be one of the best engineered models of colitis-associated cancer to dynastically study the mechanism of malignant transformation of chronic colitis.

To reveal the molecular mechanisms of colitis malignant transformation, we isolated colonic epithelial cells from the *Muc2* ^−/−^ mice and conducted miRNA profiling. We found differential expression of miRNAs at the key point of malignant transformation. Some miRNAs were characterized in human colitis and colorectal cancer tissues. Interestingly, the downregulated miRNAs were consistent with the alterations in mice, and linked to the increases of cytokines, suggesting the epigenetic alterations may play critical roles during colitis malignant transformation.

## Materials and Methods

### Ethics Statement:

The animal care and use were approved by the Institutional Animal Care and Use Committee of Xinxiang Medical University and University of Illinois at Chicago, and human samples collection and use were approved by the Institutional Review Board of Xinxiang Medical University. All patients gave informed consent in written.

### *Muc2* Mouse Model and Pathology Characterization:

As reported previously [20]–[22], the *Muc2* ^+/−^ mice were backcrossed to generate *Muc2* ^−/−^ and *Muc2* ^+/+^ mice, and 10 mice per group were fed with standard rodent chow diet for 3 months or 6 month. At the endpoints, the mice were sacrificed, entire gastrointestinal tract was opened and washed with cold PBS and fixed in 10% buffered formalin. The tissues were embedded in paraffin, sectioned and stained for histopathology characterization.

### *Muc2* Mouse Colonic Epithelia Cells Collection, mRNA Analysis, and miRNA Profiling:

Using the published protocol by us [23]–[25], mouse colonic epithelial cells were collected from 3-month aged *Muc2* ^+/+^ and *Muc2* ^−/−^ mice, respectively. Four mice from each group were used. The total RNAs were extracted using Trizol reagent (Invitrogen, Carlsbad, CA) for cytokine mRNA analysis and miRNA array analysis. The quality and quantity of the RNA was determined using Bioanalyzer and Gel electrophoresis. Cytokine mRNA levels were analyzed using q-RT-PCR. The primers used for mouse cytokine analysis were listed in Table S1.

The miRNA array was performed in the Genomic Facility of University of Chicago (Chicago, Illinois). Affymetrix GeneChip miRNA Arrays version 3.0 was used for miRNA profile. In brief, 200 ng of total RNA were labeled using FlashTag Biotin HSR Labeling Kit according manufacturer’s protocol (Affymatrix), and about 130 ul of Affymatrix hybridization cocktail buffer (FS450-002) were used for about 18 hours according to the protocol (Affymatrix). The array was then scanned using Affymatrix GeneChip Scanner 3000. The raw data was processed with Expression Console 1.2.0.20, data value was defined using Log Expression Signal - RMA-DABG. The miRNAs with a fold change >2.0 or <0.5 and a t-test value <0.01 were selected as differentially expressed miRNAs. The detailed experimental design, detailed protocol and data analysis could be accessed at Gene Expression Omnibus (GEO) (Access # GSE56577).

### Mouse miRNA Validation using quantitative Reverse Transcription Polymerase Chain Reaction (qRT-PCR):

Based on the degrees of changed miRNA levels from the miRNA array profile, 6 of the most upregulated and 9 of the most downregulated miRNAs were validated using qRT-PCR. The reverse transcription (RT) primers and forward primers and probes used for the miRNA validation were listed in Table S2. The universal Taqman probe and universal reverse primer for qRT-PCR were purchased from Integrated DNA Technologies (IDT) (Table S2).

Total RNA from mouse colonic epithelia was polyadenylated with Poly(A) Polymerase Tailing Kit (Epicentre). Briefly, 10 µl of reaction including 1 µg of RNA, 1 µl of 10x reaction buffer, 1 µl of 10 mM ATP and 1 unit of Poly(A) polymerase was incubated at 37°C for 30 minutes, followed by enzyme inactivation at 65°C for 5 min and then put on ice. After polyadenylation, reverse transcription was performed in a 10 µl reaction containing 1 µl of the polyadenylation reaction product, 1 µl of 0.5 µM RT primer, 0.5 µl of 10 mM dNTP, 1 µl of AMV 10x reaction buffer, and 50 units of AMV High Performance Reverse Transcriptase (Promega, Madison, WI). The reaction was incubated at 42°C for 60 min, and then terminated by heating at 70°C for 10 min. RT products were amplified and detected using a S-Poly(T) method, as reported by us [26]. A 20 µl PCR reaction contains 2 µl of RT products (4-fold dilution), 10 µl of 2x GoTaq® Hot Start Colorless Master Mix (Promega, Madison, WI), 0.2 µM forward primer, 0.2 µM universal reverse primer, and 0.25 µM universal Taqman probe. The PCR reaction was performed at 95°C for 30 s, followed by 40 cycles of 95°C for 10 s and 60°C for 30 s. The qRT-PCR results were analyzed as reported by us[27]–[29]. The snoRNA202 was used as internal control.

### Human Samples Collection:

Sixteen paires of human colorectal cancer tissues, colitis tissues, and their adjacent normal colon mucosa, were collected from November, 2012 through October, 2013, from the First Affiliated Hospital and the Affiliated Xinxiang Central Hospital, Xinxiang Medical University. Portion of the samples were snapped into liquid nitrogen and then stored at −80°C for RNA extraction and for qRT-PCR analysis. All patients gave informed consent in written. The sample collection and use was approved by the Institutional Review Board of Xinxiang Medical University.

RNA extraction and qRT-PCR for mRNA analysis for human samples were similar as described above for the analysis in mouse colonic epithelial cells. The primers and probes used for miRNA analysis were listed Table S2. SNORD 44 was used as internal control.

### miRNA Targets Identification, Biological Functions Categorization, and Network Analysis:

To identify the targets of the miRNAs, we used online software - David (http://david.abcc.ncifcrf.gov/) and a predicted list of conserved miRNA target genes obtained from targetscan (http://www.targetscan.org/), starBase (http://starbase.sysu.edu.cn/), Tarbase (http://microrna.gr/tarbase/), and miRbase (http://mirbase.org/index.shtml). The biological functions of the miRNAs were categorized by Gene Oncology (GO). Potent targets network and signaling were proposed using KEGG (http://www.kegg.jp) and Ensembl (http://www.ensembl.org) web tools.

## Results

### Histopathology of the *Muc2* Mouse Model of Colitis-associated Cancer:

**Figure 1:**
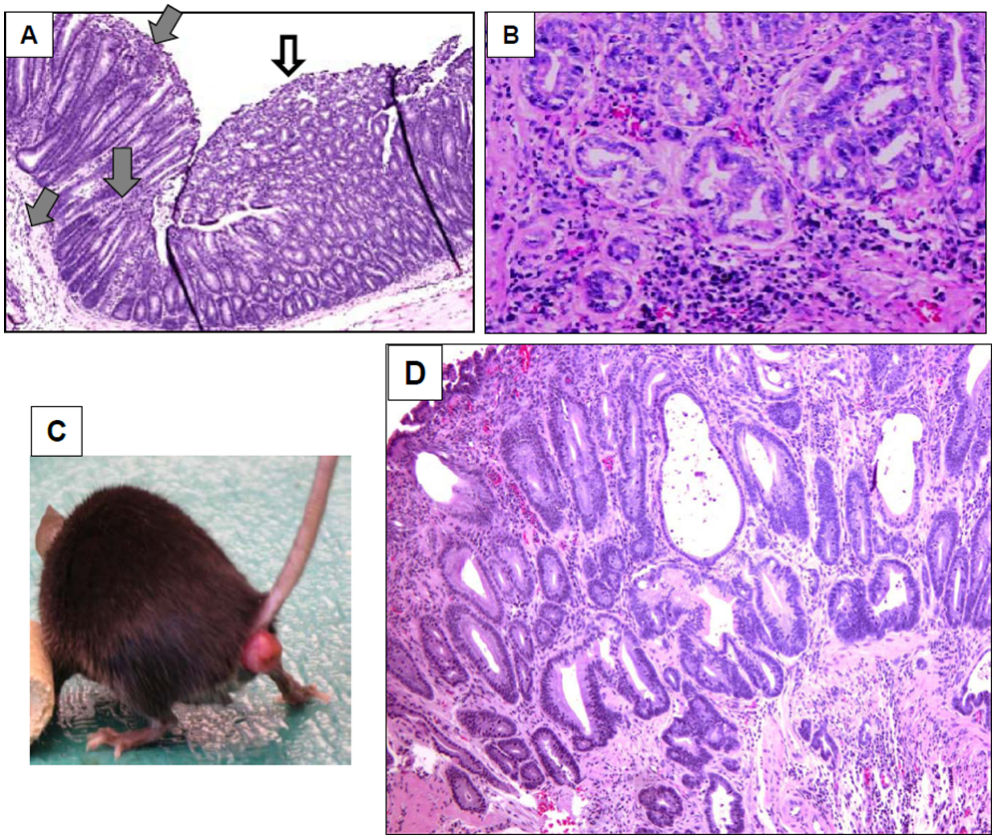
*Muc2* ^−/−^ mice exhibited chronic inflammation and cancers in colon and rectum. A, Colitis and an adenoma in mouse colon at age of 3 months. Gray arrows pointed severe infiltration of inflammatory cells, white arrow pointed an adenoma. B, colonic adenocarcinoma with inflammatory cell infiltration at age of 6 months. C, Rectal prolapsed at 6 months. D, Histopathology of the rectal prolapse, showing cancer cell invasion and severe inflammation in the rectum (age of 6 months).

Previous work have demonstrated that targeted gene knockout of the *Muc2* gene caused tumor formation in entire gastrointestinal tract, including duodenum, colon and rectum[20], and the *Muc2* ^−/−^ mice are susceptible to DSS-induced inflammatory bowl diseases[30]. In this study, we found that at 3 months or earlier, the *Muc2* ^−/−^ mice spontaneously developed chronic inflammation in colon and rectum, accompanying with a few tumors in these sites (Figure 1). As shown in Figure 1A, the colon mucosa lacked of goblet cells and exhibited the features of ulcerative colitis, such as superficial erosion and intensive infiltrations of inflammatory cells throughout the mucosa, submucosa and even muscle layer of the colon, which was similar as observed in human ulcerative colitis. The chronic colitis was accompanied by an adenoma. At the age of 6 months, *Muc2* ^−/−^ mice developed tumors at colon and rectum, as reported [20], but severe inflammation was still observed in intra-tumors and extra-tumors (Figure 1B). A unique phenotype was that *Muc2* ^−/−^ mice developed rectal prolapses (Figure 1C) that has never been reported previously. Histopathologically, the prolapse was adenocarcinoma with severe inflammation at the rectums (Figure 1D), displaying superficial erosion, inflammatory cells infiltration and cancer cell invasion. In addition, the severity of inflammatory cell infiltration was associated with the severity of rectal prolase, but was not associated with cancer cell invasion and differentiation in the rectum (data not shown).

### miRNA Profiling Revealed Differential Expression of miRNA in *Muc2^−/−^* Mice

Recent studies have suggested the important roles of miRNAs in carcinogenesis. To investigate whether aberrant expression of miRNAs are involved in colitis malignant transformation, we isolated the colonic epithelial cells from 3-month old mice (4 *Muc2* ^−/−^ mice and 4 *Muc2* ^+/+^ mice) and conducted miRNA profiling. It is known that the interaction between stromal and epithelial cells plays important role in driving colitis-associated colorectal cancer (CAC). *Muc2* was overexpressed in colonic epithelial cells, and genetic deficiency of this gene is sufficient to cause colitis and colorectal cancer, exerting the importance of *Muc2* in the development of colitis and CAC. To determine the roles of aberrantly expressed miRNAs resulted from *Muc2* absence in initiating colitis and facilitating colorectal cancer transformation, we used colonic epithelial cells instead of stromal cells or entire colon tissues for miRNA array. MiRNA profiling analysis showed differential levels of miRNAs, among them 20 miRNAs were significantly downregulated and 71 miRNAs were significantly upregulated (Table 1) in *Muc2* ^−/−^ mice, in comparison with *Muc2* ^+/+^ mice (change fold >2 or <0.5; T<0.01, p value<0.05, q value<0.05). As showing in Table S3 and Table S4, most of the miRNAs have been reported to regulate their targets and play critical roles in cancer initiation, progression and metastasis, in different tissues and cells. While, biological functions of some miRNAs are not clear and warrant further investigation.

**Table 1:** Down- and up-regulated miRNAs in *Muc2^−/−^* mouse colonic epithelial cells profiled by miRNA array.

miRNAs	Chromosome location	Mean (fold changes)	T-Test (<0.01)
**Down-regulated**			
mmu-miR-3096b-3p	–	0.053013368	0.000804092
mmu-miR-3096-3p	–	0.082183923	0.000431388
mmu-miR-146a	chr11: 43374397–43374461 [−]	0.196486214	0.005127109
mmu-miR-138	chr8: 94324311–94324381 [+]	0.247414164	0.005127109
mmu-miR-1949	chr18: 35554567–35554636 [+]	0.247414164	0.001366367
mmu-miR-5123	chr4: 40850056–40850138 [−]	0.338563887	0.009803996
mmu-miR-132	chr11: 75173682–75173747 [+]	0.362863789	0.001008516
mmu-miR-5099	chr12: 36816205–36816278 [+]	0.375009747	0.001860521
mmu-miR-1a-1-star	chr2: 180389048–180389124 [+]	0.382226577	0.001695487
mmu-miR-24-2-star	chr8: 84208815–84208921 [+]	0.396392068	0.001922905
mmu-miR-21	chr11: 86584067–86584158 [−]	0.397768242	0.001673221
mmu-miR-147	chr2: 122640803–122640881 [+]	0.401229615	0.000804292
mmu-miR-5112	chr18: 82720281–82720340 [+]	0.409660302	0.002989666
mmu-miR-677-star	chr10: 128085286–128085363 [+]	0.423372656	0.002245339
mmu-miR-150	chr7: 45121757–45121821 [+]	0.433769344	0.009595381
mmu-miR-27a	chr8: 84208672–84208758 [+]	0.470576115	0.000890949
mmu-miR-196b-star	chr6: 52230081–52230165 [−]	0.48380581	0.006710287
mmu-miR-23a	chr8: 84208518–84208592 [+]	0.498270131	0.001669314
mmu-miR-145	chr18: 61647825–61647894 [−]	0.498570581	0.001129314
mmu-miR-5105	–	0.49933846	0.000289965
**Up-regulated**			
mmu-miR-466g	chr2: 10514595–10514674 [+]	2.0139111	0.000615992
mmu-miR-327	chr14: 44947443–44947511 [−]	2.056227653	0.003244991
mmu-miR-466m-3p	chr2: 10466663–10466746 [+]	2.077718207	0.00355787
mmu-miR-466f-3p	–	2.084931522	0.003418186
mmu-miR-1931	chr10: 93162785–93162903 [+]	2.088547565	0.000362476
mmu-miR-1894-3p	chr17: 35917889–35917969 [+]	2.095798477	0.002599441
mmu-miR-3470b	chr16: 44013852–44013977 [+]	2.110375908	0.006057193
mmu-miR-1896	chr13: 21445160–21445240 [+]	2.199994627	0.004807219
mmu-miR-3093-3p	chr3: 88215171–88215257 [+]	2.230705407	0.004988571
mmu-miR-195-star	chr11: 70235042–70235135 [+]	2.281527432	0.006474418
mmu-miR-204-star	chr19: 22750605–22750672 [+]	2.317388619	0.004938058
mmu-miR-125a-3p	chr17: 17830812–17830879 [+]	2.321407829	0.002948655
mmu-miR-669f-3p	chr2: 10467229–10467349 [+]	2.325434009	0.002838976
mmu-miR-16-1-star	chr14: 61631880–61631972 [−]	2.374296321	0.008688873
mmu-miR-466i-3p	chr13: 17747473–17747593 [+]	2.411615655	0.002526037
mmu-miR-1943	chr15: 79375228–79375300 [−]	2.428389769	0.002672958
mmu-miR-1967	chr8: 124022641–124022722 [+]	2.441046876	0.005668811
mmu-miR-150-star	chr7: 45121757–45121821 [+]	2.470837274	0.008970092
mmu-miR-383	chr8: 38252133–38252202 [−]	2.505328877	5.02E-05
mmu-miR-669p-star	chr2: 10489116–10489202 [+]	2.549121255	0.003814622
mmu-miR-877	chr17: 35960730–35960814 [−]	2.553542375	0.008090848
mmu-miR-92a-2-star	chrX: 52741838–52741928 [−]	2.611719574	0.004892665
mmu-miR-669c-star	chr2: 10509296–10509404 [+]	2.634446716	0.000446431
mmu-miR-5133	chr9: 62122518–62122594 [−]	2.643592852	0.001549712
mmu-miR-3113-star	chrX: 151859562–151859637 [+]	2.727350278	0.001069083
mmu-miR-3082-5p	chr17: 25831365–25831428 [−]	2.74156561	0.001627678
mmu-miR-466i-5p	chr13: 17747473–17747593 [+]	2.784657705	0.005962544
mmu-miR-574-5p	chr5: 64970318–64970395 [+]	2.789487333	0.001972081
mmu-miR-3474	chr2: 158638583–158638640 [+]	2.923101651	0.004805635
mmu-miR-466h-3p	chr2: 10514891–10514971 [+]	2.933249925	0.001659026
mmu-miR-5120	chr4: 44607491–44607568 [−]	3.010493495	0.003511011
mmu-miR-467d-star	chr2: 10507630–10507714 [+]	3.08977118	0.004977926
mmu-miR-546	chr10: 126998440–126998560 [+]	3.116658319	0.000267697
mmu-miR-615-5p	chr15: 103014910–103015001 [+]	3.165646144	0.001906752
mmu-miR-466j	chr10: 60960723–60960844 [+]	3.193193545	0.00044491
mmu-miR-466h-5p	chr2: 10514891–10514971 [+]	3.215403963	0.003518948
mmu-miR-1962	chr7: 135566162–135566282 [+]	3.305801273	0.000199144
mmu-miR-296-3p	chr2: 174267047–174267125 [−]	3.369419364	0.002478334
mmu-miR-667-star	chr12: 109720006–109720097 [+]	3.369419364	0.005918912
mmu-miR-135a-1-star	chr9: 106154124–106154213 [+]	3.392855529	0.002717844
mmu-miR-669f-5p	chr2: 10467229–10467349 [+]	3.422380103	0.005286193
mmu-miR-5110	chr11: 85760616–85760702 [−]	3.452161599	0.00719303
mmu-miR-5122	chr4: 133369776–133369864 [+]	3.464146636	0.001568357
mmu-miR-3075	chr14: 25534439–25534523 [+]	3.512504321	0.000262538
mmu-miR-1930-star	chr10: 77641224–77641307 [+]	3.543070076	0.000277333
mmu-miR-328-star	chr8: 105308364–105308460 [−]	3.555370725	0.006676725
mmu-miR-669c	chr2: 10509296–10509404 [+]	3.573901815	0.000129374
mmu-miR-3064-5p	chr11: 106782693–106782759 [−]	3.586309503	0.001181755
mmu-miR-129-5p	chr6: 29022619–29022691 [+]	3.61751751	0.000610483
mmu-miR-466f	chr2: 10466944–10467037 [+]	3.642679334	0.001960095
mmu-miR-92b-star	chr3: 89227116–89227198 [−]	3.655325801	0.000962434
mmu-miR-3473	chrX: 162874918–162874995 [−]	3.738604865	0.005536237
mmu-miR-211-star	chr7: 64205806–64205911 [+]	3.837056477	0.00152237
mmu-miR-760-3p	chr3: 122293585–122293703 [−]	3.9244759	0.002240405
mmu-miR-669n	chr3: 115979902–115979955 [+]	4.17709513	0.009992096
mmu-miR-704	chr6: 47803576–47803652 [−]	4.25748073	0.002715333
mmu-miR-3470a	chr6: 83090311–83090387 [−]	4.399989253	0.002512862
mmu-miR-1966	chr8: 105615466–105615573 [+]	4.407620464	0.000453366
mmu-miR-323-5p	chr12: 109712508–109712593 [+]	4.563054863	0.00046665
mmu-miR-3104-5p	chr7: 141992179–141992241 [+]	4.667014663	0.000569568
mmu-miR-3102-star	chr7: 100882306–100882409 [−]	4.699476317	0.001673908
mmu-miR-3081-star	chr16: 44558046–44558129 [−]	4.83159658	0.000792041
mmu-miR-5130	chr14: 102982549–102982632 [−]	5.445256466	0.001466623
mmu-miR-3472	−	6.158162767	0.006037372
mmu-miR-3077-star	chr14: 57798424–57798487 [+]	6.531887757	0.008639499
mmu-miR-1982-star	chr10: 80828797–80828870 [+]	6.680703355	0.006823859
mmu-miR-5135	chr12: 76533134–76533212 [−]	7.727490631	0.004441672
mmu-miR-705	chr6: 85336292–85336373 [−]	7.8489518	0.000703155
mmu-miR-5132	chrX: 74023528–74023598 [−]	11.95879399	0.006610448
mmu-miR-5115	−	16.16722314	0.008489482
mmu-miR-5102	−	21.00265778	0.003512181

### Mouse miRNAs Validation by qRT-PCR:

**Figure 2:**
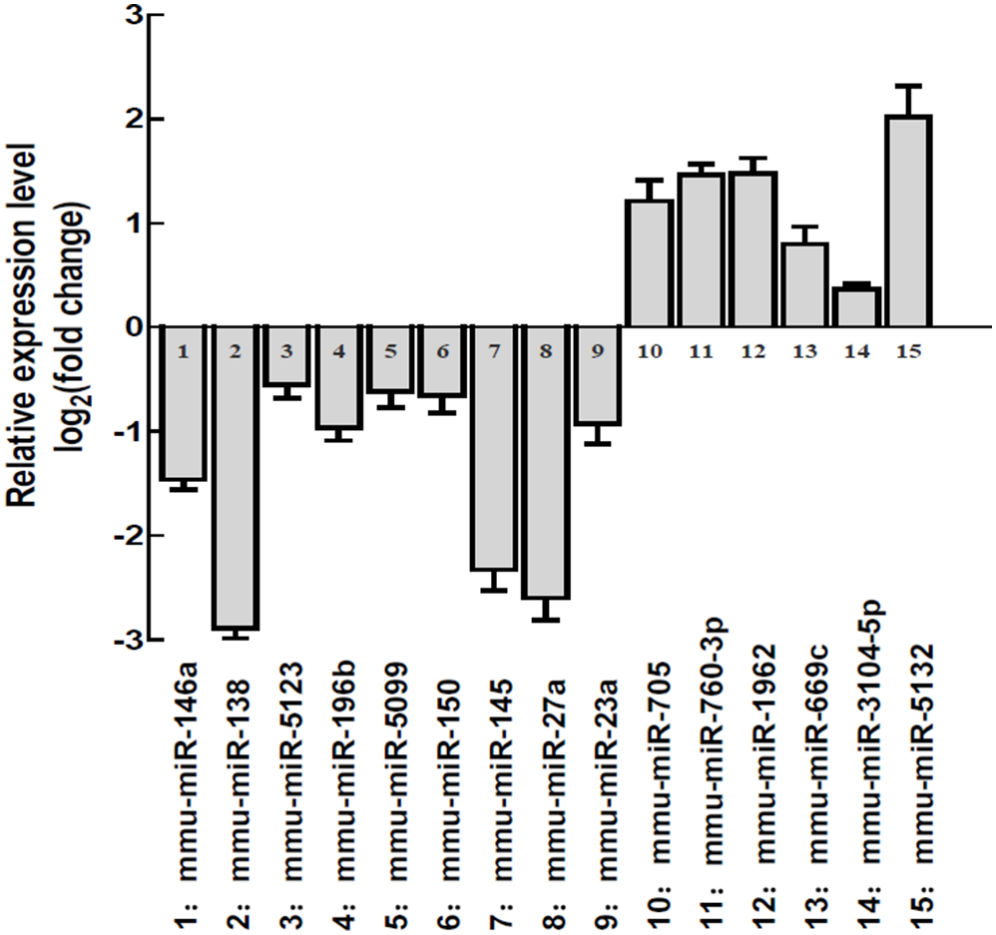
The aberrantly expressed miRNAs were validated in *Muc2* ^−/−^ mice colonic epithelial cells, in comparison with *Muc2* ^+/+^ mice. Four mice at age of 3 months from each genotype group were sacrificed and colonic epithelial cells were isolated for RNA extraction and qRT-PCR analysis. The columns stood for Mean+/−SD.

To evaluate the accuracy of the profiled miRNAs alteration in mouse colonic epithelial cells, we selected 15 most relevant miRNAs for validation using qRT-PCR. The 6 upregulated miRNAs (mmu-miR-5132-5p, mmu-miR-3104-5p, mmu-miR-669c-5p, mmu-miR-705, mmu-miR-760-3p, mmu-miR-1962) and the 9 downregulated miRNAs (mmu-miR-146a, mmu-miR-138, mmu-miR-5123, mmu-miR-196b, mmu-miR-5099, mmu-miR-150, mmu-miR-145, mmu-miR-27a, mmu-miR-23a) chosen for validation were also based on their target genes predicted, whose functions are well relevant to inflammation and cancer. As shown in Figure 2, the changes of miRNA assayed by qRT-PCR were consistent with the changes profiled by miRNA array analysis.

### Aberrant Expression of miRNAs in Human Colitis, Colorectal Tumor and Adjacent Normal Mucosa

**Figure 3:**
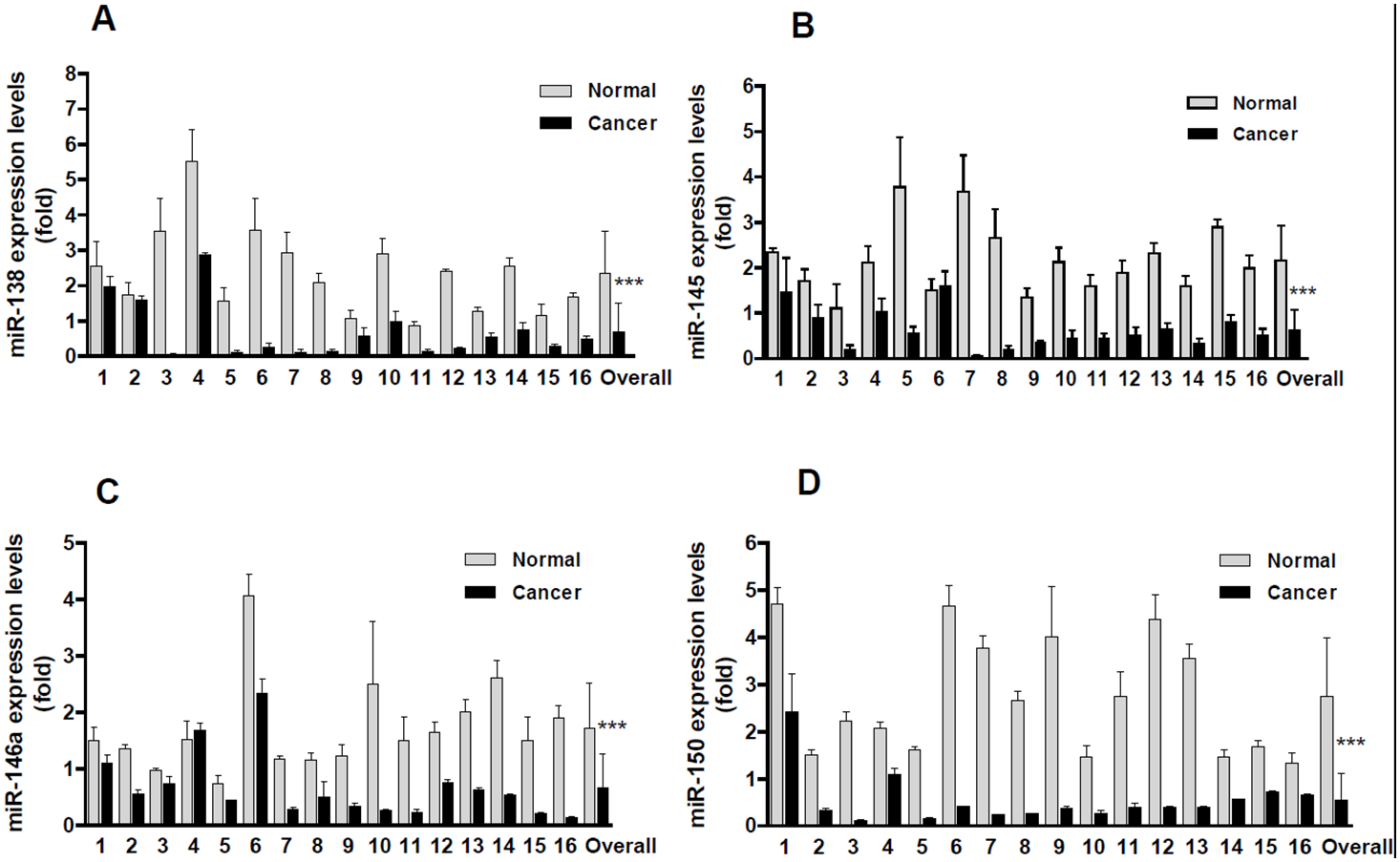
The downregulated miRNAs were confirmed in human colorectal cancers. A, miR-138 was significantly downregulated in colorectal cancers. B, miR-145 was significantly downregulated in colorectal cancers. C, miR-146a was significantly downregulated in colorectal cancers. D, miR-150 was significantly downregulated in colorectal cancers. Each column stood for one patient, total 16 patients. The Overall stood for the Mean of the miRNAs in all patients. (***p<0.0001, compared to adjacent normal mucosa).

**Figure 4:**
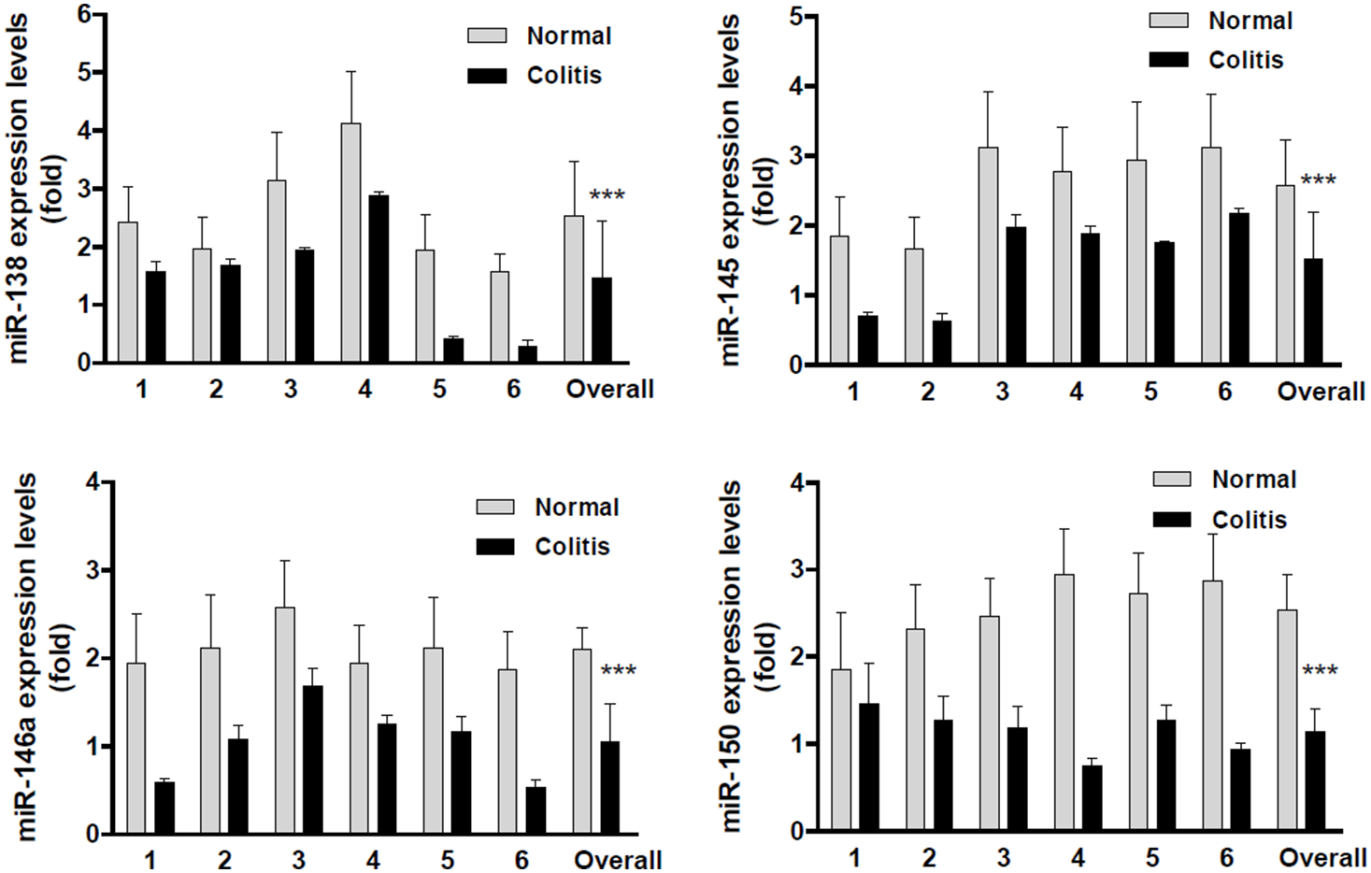
The downregulated miRNAs were confirmed in human colitis. A, miR-138 was significantly downregulated in colitis. B, miR-145 was significantly downregulated in colitis. C, miR-146a was significantly downregulated in colitis. D, miR-150 was significantly downregulated in colitis. Each column stood for one patient, total 6 patients. The Overall stood for the Mean of the miRNAs in all patients. (***p<0.0001, compared to adjacent normal mucosa).

To determine whether the aberrantly expressed miRNAs have clinical significance, we chose 4 miRNAs for analysis in human colorectal cancer and colitis tissues. As shown in Figure 3, in overall, the expression level of miR-138, miR-145, miR-146a and miR-150 were downregulated by approximately 3.37, 3.39, 2.56 and 4.99 fold in colorectal cancers than those in the matched adjacent normal mucosa (*p*<0.0001). Among them, all the 16 colorectal cancers showed downregulated miR-138 and miR-150 levels (Figures 3A and 3D), and 15 out of the 16 colorectal cancers showed lower miR-145 and miR-146a expression levels than normal control (Figures 3B and 3C). Only one patient (Sample 6) showed upregulation of miR-145, but the upregulation was not significant (Figure 3B, p>0.05). Interestingly, the downregulation of these miRNAs were also observed in human chronic colitis tissues (Figures 4, p<0.0001, compared to the normal mucosa). Although the observations were obtained from small sized samples, the trends of significant downregulation of miRNAs (miR-138, 145, 146a and miR-150) strongly suggested their clinical importance of linkage to chronic colitis and colitis-associated colorectal cancer, indirectly indicating their potential biological functions of involving in colitis malignant transformation. In fact, the functions of these miRNAs on tumor suppression are being under investigation using manipulated cell culture system *in vitro* and tumor-bearing nude mice *in vivo*.

### miRNA Target Network Characterization:

**Figure 5:**
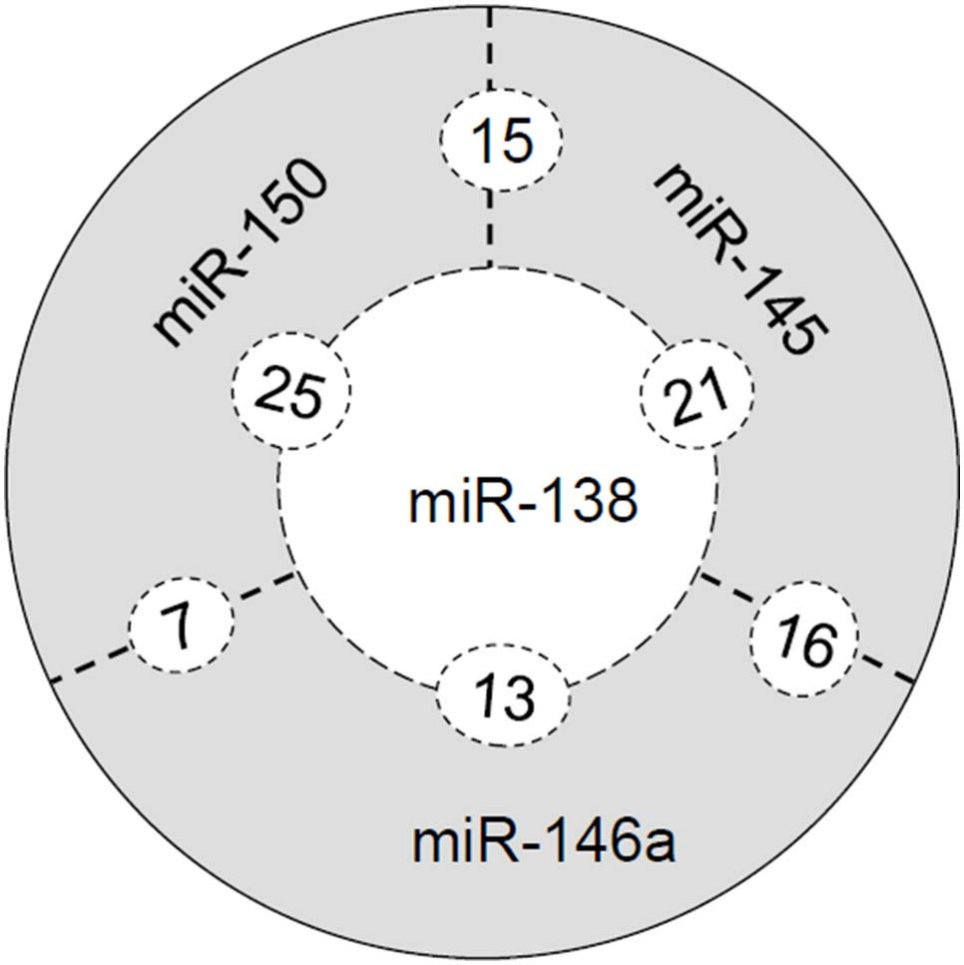
Numbers of the common targets of the miRNAs (miR-138, 145, 146a and miR-150), and their network.

Since the changed miRNAs had great significance in *Muc2* ^−/−^ mice and human colitis malignant transformation, and the selected miRNA showed downregulation in colitis and colorectal cancers, we next elucidated whether there were any common targets of these miRNAs. Unfortunately, no common inflammation- and cancer-associated targets for all of the 4 miRNAs (miR-138, 145, 146a and miR-150) were identified using miRNA target prediction tools. However, we did find some common targets of any 3 or 2 of the 4 miRNAs. As shown in Figure 5 and Table 2, there were 21, 13 and 25 common targets between miR-138 and miR-145, miR-146a and miR-150, respectively; there were 16 and 15 common between miR-145 and miR-146a and miR-150, respectively; and there were 7 common targets between miR-146a and miR-150. Please be noted that CCT3 and PAPPA were the common targets for miR-138, miR-146a and miR-150, and ZHX2 was the common target for miR-138, miR-145 and miR-150. Interestingly, most of these targets are oncogenes. GO term annotation showed that all the targets are involved in cellular physiological process and metabolism, and the regulation of cellular process and regulation of physiological process are the most significantly enriched GO terms. Thus, any dysregulation of these miRNAs and their targets might be sufficient to cause initiation of inflammation and chronic inflammation malignant transformation, studies on any common targets of two or more miRNAs in carcinogenesis is more significant than any targets of a single miRNA.

**Table 2:** Predicted common targets of 2 miRNAs (Names in bold are the common targets for at least 3 miRNAs).

miR-138 and −145	miR-138 and −146a	miR-145 and −146a	miR-138 and −150	miR-145 and −150	miR-150 and −146a
BAG1	BCL11A	ARPC5	**CCT3**	ACBD3	BZRAP1
CCDC107	BUD13	BSG	DHDDS	AMOTL2	**CCT3**
CLK3	**CCT3**	C20orf20	DVL2	BSN	IRAK1
CPEB4	CD1B	CD53	EIF4EBP1	C1orf177	NPTN
DUSP6	DMKN	DYX1C1	ELF2	CCDC88	**PAPPA**
FBXO18	MAT2A	CCPG1	ERC1	DYRK1A	SMC3
FMNL2	MXD4	FBXL10	LRRC4	EIF4B	TRAF2
GGTL3	**PAPPA**	GAD1	MAP3K11	EIF4EBP2	
GPHN	PDLIM1	HEMGN	MYBBP1A	MBNL1	
INHBB	PHF1	HIC2	NKIRAS2	NPM3	
LENG8	PHOX2B	IVNS1ABP	**PAPPA**	SIRT5	
MAP3K6	RUNX1T1	PCBP2	PFN2	SMARCD1	
MECP2	VIM	STC1	PPARGC1A	STK19	
PHF21A		TLN2	PTP4A1	TMEM116	
PITX3		VASN	PURB	**ZHX2**	
RARA		ZFYVE9	RAD23B		
RBM41			RAI17		
S100A2			SEH1L		
WFDC11			SUMO1		
**ZHX2**			TP53INP2		
ZMYND11			TRPS1		
			UPF2		
			VEGFB		
			ZBTB		
			**ZHX2**		

### Cytokines in *Muc2^−/−^* Mouse Colonic Epithelial Cells were Up-regulated:

**Figure 6:**
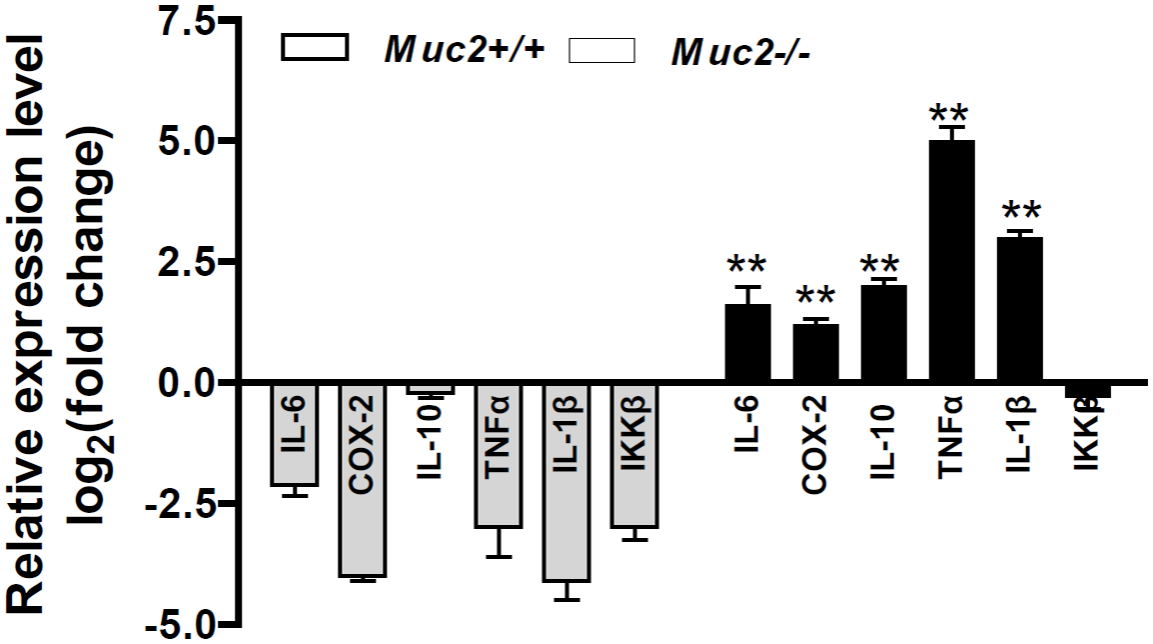
The alterations of cytokines in *Muc2* mouse colonic epithelial cells. Compared to *Muc2* ^+/+^ mice, *Muc2* ^−/−^ mice showed significant upregulation of cytokines (**p<0.01, compared to *Muc2* ^+/+^ mice). Four mice at age of 3 months from each genotype group were sacrificed and colonic epithelial cells were isolated for RNA extraction and qRT-PCR analysis. The columns stood for Mean+/−SD.

To validate the accuracy of the analysis of the miRNA and their association with cytokine mRNAs, the later are frequently seen in colitis-associated cancer, we determine the alterations of cytokine in mouse colon. As shown in Figure 6, compared to *Muc2* ^+/+^ mouse, *Muc2* ^−/−^ mice showed significant upregulation of cytokines (e.g. IL-6, Cox2, TNF-a, and IL-1β, etc) in colonic epithelial cells from the normal appearing colon mucosa (p<0.01).

## Discussion

The current study further characterized a colitis-associated colorectal cancer (CAC) model of *Muc2* ^−/−^ mice, providing direct evidence that chronic inflammation in colon and rectum could be malignantly transformed, and revealing the potent mechanisms, that was that aberrant miRNA expression in colonic epithelial cells were involved in and might play critical roles during the malignant transformation of chronic colitis by interfering target genes.

Numerous evidence-based studies have demonstrated that chronic colitis is one of the important causes of colorectal cancer, but the underlying mechanisms are not clear, one of the major reasons is lack of spontaneous colitis and colitis-associated colorectal cancer model. Currently, a variety of genetically engineered animal models are available and are very useful for better understanding of the molecular mechanisms underlying the pathogenesis of CAC [31], but pathological features of these models have not been dynamically characterized. The present study characterized the *Muc2* mouse model. The *Muc2* ^−/−^ mice spontaneously develop chronic inflammation in colon and rectum at early stage (<3 months), and after 3 months, the chronic inflammation progress to colorectal cancer. Most importantly, the pathologic features of the chronic inflammation at colon and rectum were similar as human ulcerative colitis, and the aberrantly expressed miRNAs involved in *Muc2* ^−/−^ mouse colitis malignant transformation were observed in human colitis and colorectal cancer tissues. Another unique feature was the rectal prolapses – rectal cancer with severe inflammation. Therefore, the *Muc2* ^−/−^ mouse could be the appropriate rodent model of CAC, and could be used as one of the best tools to elucidate the cause of CAC and the underlying molecular mechanisms. The *Muc2* ^−/−^ mouse could also be used as a mouse model for chemoprevention and therapy for colitis-associated colorectal cancer.

miRNAs have 19–22 nucleotides, are a novel class of small noncoding RNAs that suppress the translation and stability of messenger RNAs (mRNAs) by binding to target mRNAs’ 3′-untranslated regions (3′-UTR)[32], miRNAs have important biological and pathophysical functions of involving in development, cell proliferation, differentiation, apoptosis, inflammation and stress response[33]–[35]. Increasing evidences have suggested that miRNAs are downregulated or upregulated in cancers, acting as tumor suppressors or oncogenes [33], [35], [36], in which the miRNAs play critical roles in tumorigenesis, differentiation, progression (e.g. migration, invasion, angiogenesis, and metastasis) [35]–[37], mainly by interfering with the expression of target genes. Using a miRNA array, we have profiled differential expression of miRNAs in colonic epithelial cells from the *Muc2* ^−/−^ mice, and these miRNAs are either upregulated as oncogenes or downregulated as tumor suppressors by targeting the genes at the categories of metabolism, cell cycle, differentiation, cell death, DNA replication, homeostasis, signal transduction, response to stimulation, and inflammation, etc. Among the markedly changed miRNAs, the downregulated miRNAs, miR-138, 145, 146a and 150, were validated in both mouse and human tissues, particularly, in human colitis and colorectal cancer tissues, suggesting suppressing roles of miR-138, 145, 146a and 150 in colitis malignant transition via interacting with cytokines and inflammatory factors.

Previous studies have demonstrated tumor suppressor roles of miR-138 in cancer biology. miR-138 inhibited cancer cell growth and tumorigenesis in non-small cell lung cancer and nasopharyngeal cancer by targeting 3-phosphoinositide-dependent protein kinase-1 (PDK1) and CCND1 [38]–[40]. In colorectal and ovarian cancers, miR-138 suppressed cancer cell migration and metastasis through interfered with TWIST2, SOX4 and HIF1-a [39], [41]. Most recent studies reported that downregulated miR-138 sustained inflammatory factor NF-kB activation and promoted esophageal cancer progression [42], and that miR-138 response to pro-inflammatory cytokines depends on the stabilization of HIF1-α in primary human microvascular endothelial cells [43]. miR-145 is also a tumor suppressor gene. miR145 could target the SOX9/ADAM17 axis and inhibit tumor-initiating cells and IL-6-mediated paracrine effects in head and neck cancer[44]. Moreover, microRNA-145 induces apoptosis with the induction of tumor necrosis factor-related apoptosis-inducing ligand (TRAIL) expression, targeted oncogene socs7 and regulated interferon-β induction through STAT3 nuclear translocation in bladder cancer cells [45]. Recent studies have reported that TRAIL suppresses chemokine (C-X-C motif) receptor 4 (CXCR4) -mediated human breast cancer cell migration by up-regulating miR-146a expression through NF-κB signaling [46], and that miR-146 regulates epigenetic regulator UHRF1 and modulates gastric cancer invasion and metastasis [47], showing the important roles of miR-146 in inhibiting cancer metastasis by interfering chemokine and epigenetic regulator. As to miR-150, quite a lot of reports have demonstrated its tumor inhibitory function [48]–[51]. While, that miR-150 interacts with cytokines in lymphocyte differentiation and in inflammation has been studied. For instance, in cytotoxic T lymphocytes (CTL), IL-2R and inflammatory signals act through Dicer and miRNAs to control the cytolytic program and CTL differentiation, in which miR-139 and miR-150 are downregulated by inflammation in CTLs, and miR-150 regulates the expression of the IL-2 receptor α-chain (CD25) [52]. In addition, IFN-γ production is significantly increased in the miR-150 knockout mice [53]. Back to our findings, that were, cytokines were significantly increased (Figure 6) and miR-138, 145, 146a and miR-150 were significantly decreased in *Muc2* ^−/−^ mouse colon and human colitis and colorectal cancer, incorporating with the published observations, strongly support our hypothesis that the cytokine-associated miRNAs, miR-138, 145, 146a and miR-150, play important roles in chronic colitis malignant transformation through interfering with cytokines and inflammatory factors. However, similar as the changes of cytokines as a consequence of colitis in mouse colon, the changes of the miRNAs could also be a consequence chronic colitis and CAC in human colon tissues. It could be possible, but the preliminary data from our ongoing functional studies using manipulated cell culture systems and in vivo nude mouse model have shown individual or synergistical potential of these miRNA (Bao and Yang, unpublished data), confirming tumor suppressing functions of these miRNAs. In addition, the potent resources of the altered miRNAs in human colitis and CRC tissues are not clear and need further investigation, the restults generated from which could clarify the functions cause or effect of these miRNAs in the development of colitis and colorectal cancer.

Our study further identified some common targets of the miR-138, 145, 146a and miR-150 (Table 2 and Figure 5), such as PAPPA (pregnancy-associated plasma protein A), CCT3 (chaperonin containing TCP1, subunit 3) and ZHX2 (zinc fingers and homeoboxes 2), which were the common targets of three miRNAs. These three targets have been reported to be overexpressed in embryonic cells and stem cells, and exert oncogenic functions during cell process and proliferation [54]–[56], which could play synergistic roles with miRNA-regulated cytokine activation to initiate colorectal cancer formation and facilitate colorectal cancer progression. These targets’ expression levels in normal mucosa, colitis and colorectal cancer tissues are not clear, and their biological functions in cancer, are not clear and under investigation. Moreover, it is worthy to point out that tumor formation and progression are controlled by a complex not by a single element, thus, studying on multiple miRNAs and multiple targets, especially on common targets of multiple miRNAs, instead of studying on a single target or single miRNA, are more important, to reveal the causes of cancers and the molecular mechanisms.

Taken above, we have characterized a colitis-associated colorectal cancer model, and revealed the underlying mechanisms that aberrant expressed miRNAs targets cytokines and downstream genes to facilitate colitis malignant transformation. This is the first to reveal the importance of aberrant expression of miRNAs in dynamically transformation from chronic colitis to colitis-associated cancer. These findings shed light on revealing the mechanisms of chronic colitis malignant transformation.

## Supporting Information

Table S1 **Primer sequences for the mouse qRT-PCR.**
(DOCX)

Table S2 **Primers and Probes for miRNA qRT-PCR.**
(XLSX)

Table S3 **Oncogenic miRNAs upregulated in Muc2** ^−/−^ **mouse colonic epithelial cells profiled by miRNA** array.
(DOC)

Table S4 **Tumor suppressor miRNAs downregulated in Muc2** ^−/−^ **mouse colonic epithelial cells profiled by** **miRNA array.**
(DOC)
