# Supplementary material for: MicroRNA Profiling in Muc2 Knockout Mice of Colitis-Associated Cancer Model Reveals Epigenetic Alterations during Chronic Colitis Malignant Transformation
Source: PLoS One. 2014 Jun 18;9(6):e99132. doi: 10.1371/journal.pone.0099132 (PMC4062425; doi:10.1371/journal.pone.0099132)
Supplement: Table S1 — Primer sequences for the mouse qRT-PCR. (DOCX) [file pone.0099132.s001.docx]

**Support information**

**Bao, et al**

MicroRNA profiling in Muc2 mice of colitis-associated cancer model reveals epigenetic alterations during chronic colitis malignant transformation

**Table S1 Primer sequences for the mouse qRT-PCR**

**Gene name Forward-primer sequences Reverse-primer sequences**

IL-6 TAGTCCTTCCTACCCCAATTTCC TTGGTCCTTAGCCACTCCTTC

COX-2 TGAGCAACTATTCCAAACCAGC GCACGTAGTCTTCGATCACTATC

IL-10 GCTCTTACTGACTGGCATGAG CGCAGCTCTAGGAGCATGTG

IL-1β GCAACTGTTCCTGAACTCAACT ATCTTTTGGGGTCCGTCAACT

TNFα CCCTCACACTCAGATCATCTTCT GCTACGACGTGGGCTACAG

IKKβ CTGAAGATCGCCTGTAGCAAA TCCATCTGTAACCAGCTCCAG

GAPDH TCTGGAAAGCTGTGGCGTGAT GCCAGTGAGCTTCCCGTTCAG

_______________________________________________________________________________
