# Supplementary material for: MicroRNA Profiling in Muc2 Knockout Mice of Colitis-Associated Cancer Model Reveals Epigenetic Alterations during Chronic Colitis Malignant Transformation
Source: PLoS One. 2014 Jun 18;9(6):e99132. doi: 10.1371/journal.pone.0099132 (PMC4062425; doi:10.1371/journal.pone.0099132)
Supplement: Table S3 — Oncogenic miRNAs upregulated in Muc2 −/− mouse colonic epithelial cells profiled by miRNA array. (DOC) [file pone.0099132.s003.doc]

**Table S3**

**Oncogenic miRNAs upregulated in Muc2-/- mouse colonic epithelial cells**

profiled by miRNA array

| **microRNA** | **Tumor/Cell line** | **Target** | **Notes** | **References** |
| --- | --- | --- | --- | --- |
| miR-129-5p | Laryngeal  squamous cell  carcinoma | APC | miR-129-5p targeted APC to release inhibition of Wnt signalling | [1] |
| miR-574-5p | human lung cancer | Ches1 | Ches1 as the dominant direct target for miRNA-574-5p to confer the TLR9 signaling enhanced tumor progression | [2] |
| miR-296-3p | Prostate cancer | ICAM-1 | MiRNA-296-3p-ICAM-1 axis promotes metastasis of prostate cancer | [3] |
| miR-135a | colorectal cancer | APC | miR-135a suppress APC expression and induce downstream Wnt pathway activity | [4] |
| miR-92b-star | NSCLC cell | RECK | miR-92b might promote NSCLC cell growth and motility partially by inhibiting RECK | [5] |
|  | non-small cell  lung cancer | PTEN | miR-92b plays an oncogene roles, acting as a novel potential maker for NSCLC therapy | [6] |
|  | liver cancer stem cells | C/EBPß | miR-92b can result inproliferation increase and differentiation arrest of hepatic progenitors | [7] |
| miR-23a | Glioma | HOXD10 | miRNA-23a promoted U251 and U87 cell invasion, | [8] |
| miR-21 | colorectal  cancer | ITGβ4 | ITGβ4 is a novel miR-21 target gene and plays a role in the regulation of EMT | [9] |
| miR-92a-2-star | small cell lung cancer  glioblastoma | PHLPP2  PI3K/Akt | Higher miR-92a-2* levels are associated with chemoresistance and with decreased survival in patients  miR-92a targets PHLPP2 and promotes tumorigenesis and metastasis | [10][11] |
| miR-3470b | lung cancer  breast cancer | Cont2 | Mir3470b is a likely regulator of the Cnot2 network | [12] |
| miR-323-5p | glioblastoma | - | low levels of miR-323/miR-329/miR-155/miR-210 were significantly associated with long OS of GBM patients | [13] |
| miR-3470a | lung cancer  breast cancer | Cont2 | Mir3470a/b promote metastasis | [12] |
| miR-150 | lung cancer | SRCIN1 | the repression of SRCIN1 by miR-150 consequently triggered the activation of FAK and ERK pathway | [14] |

**References**:

1. Li, M., Tian, L., Wang, L., Yao, H., Zhang, J., Lu, J., Sun, Y., Gao, X., Xiao, H. and Liu, M. (2013) Down-regulation of miR-129-5p inhibits growth and induces apoptosis in laryngeal squamous cell carcinoma by targeting APC. *PLoS One*, **8**, e77829.

2. Li, Q., Li, X., Guo, Z., Xu, F., Xia, J., Liu, Z. and Ren, T. (2012) MicroRNA-574-5p was pivotal for TLR9 signaling enhanced tumor progression via down-regulating checkpoint suppressor 1 in human lung cancer. *PLoS One*, **7**, e48278.

3. Liu, X., Chen, Q., Yan, J., Wang, Y., Zhu, C., Chen, C., Zhao, X., Xu, M., Sun, Q., Deng, R., Zhang, H., Qu, Y., Huang, J., Jiang, B. and Yu, J. (2013) MiRNA-296-3p-ICAM-1 axis promotes metastasis of prostate cancer by possible enhancing survival of natural killer cell-resistant circulating tumour cells. *Cell Death Dis*, **4**, e928.

4. Nagel, R., le Sage, C., Diosdado, B., van der Waal, M., Oude Vrielink, J.A., Bolijn, A., Meijer, G.A. and Agami, R. (2008) Regulation of the adenomatous polyposis coli gene by the miR-135 family in colorectal cancer. *Cancer Res*, **68**, 5795-802.

5. Lei, L., Huang, Y. and Gong, W. (2013) Inhibition of miR-92b suppresses nonsmall cell lung cancer cells growth and motility by targeting RECK. *Mol Cell Biochem*, **387**, 171-6.

6. Li, Y., Li, L., Guan, Y., Liu, X., Meng, Q. and Guo, Q. (2013) MiR-92b regulates the cell growth, cisplatin chemosensitivity of A549 non small cell lung cancer cell line and target PTEN. *Biochem Biophys Res Commun*, **440**, 604-10.

7. Qian, N.S., Liu, W.H., Lv, W.P., Xiang, X., Su, M., Raut, V., Chen, Y.L. and Dong, J.H. (2013) Upregulated microRNA-92b regulates the differentiation and proliferation of EpCAM-positive fetal liver cells by targeting C/EBPss. *PLoS One*, **8**, e68004.

8. Hu, X., Chen, D., Cui, Y., Li, Z. and Huang, J. (2013) Targeting microRNA-23a to inhibit glioma cell invasion via HOXD10. *Sci Rep*, **3**, 3423.

9. Ferraro, A., Kontos, C., Boni, T., Bantounas, I., Siakouli, D., Kosmidou, V., Vlassi, M., Spyridakis, Y., Tsipras, I., Zografos, G. and Pintzas, A. (2013) Epigenetic regulation of miR-21 in colorectal cancer: ITGB4 as a novel miR-21 target and a three-gene network (miR-21-ITGBeta4-PCDC4) as predictor of metastatic tumor potential. *Epigenetics*, **9**.

10. Ranade, A.R., Cherba, D., Sridhar, S., Richardson, P., Webb, C., Paripati, A., Bowles, B. and Weiss, G.J. (2010) MicroRNA 92a-2*: a biomarker predictive for chemoresistance and prognostic for survival in patients with small cell lung cancer. *J Thorac Oncol*, **5**, 1273-8.

11. Li, M., Guan, X., Sun, Y., Shu, X. and Li, C. (2014) miR-92a family and their target genes in tumorigenesis and metastasis. *Exp Cell Res*, pii: S0014-4827(13)00560-0. doi: 10.1016/j.yexcr.2013.12.025. [Epub ahead of print].

12. Faraji, F., Hu, Y., Wu, G., Goldberger, N.E., Walker, R.C., Zhang, J. and Hunter, K.W. (2014) An integrated systems genetics screen reveals the transcriptional structure of inherited predisposition to metastatic disease. *Genome Res*, **24**, 227-40.

13. Qiu, S., Lin, S., Hu, D., Feng, Y., Tan, Y. and Peng, Y. (2013) Interactions of miR-323/miR-326/miR-329 and miR-130a/miR-155/miR-210 as prognostic indicators for clinical outcome of glioblastoma patients. *J Transl Med*, **11**, 10.

14. Cao, M., Hou, D., Liang, H., Gong, F., Wang, Y., Yan, X., Jiang, X., Wang, C., Zhang, J., Zen, K., Zhang, C.Y. and Chen, X. (2014) miR-150 promotes the proliferation and migration of lung cancer cells by targeting SRC kinase signalling inhibitor 1. *Eur J Cancer*.
