# Supplementary material for: MicroRNA Profiling in Muc2 Knockout Mice of Colitis-Associated Cancer Model Reveals Epigenetic Alterations during Chronic Colitis Malignant Transformation
Source: PLoS One. 2014 Jun 18;9(6):e99132. doi: 10.1371/journal.pone.0099132 (PMC4062425; doi:10.1371/journal.pone.0099132)
Supplement: Table S4 — Tumor suppressor miRNAs downregulated in Muc2 −/− mouse colonic epithelial cells profiled by miRNA array. (DOC) [file pone.0099132.s004.doc]

**Table S4**

**Tumor suppressor miRNAs downregulated in Muc2-/- mouse colonic epithelial cells**

**profiled by miRNA array**

| **microRNA** | **Tumor/Cell line** | **Targets** | **Notes** | **References** |
| --- | --- | --- | --- | --- |
| miR-204-star | intrahepatic cholangio-  carcinoma | slug | miR-204 plays negative roles in the invasive and/or metastatic potential of ICC, and that its suppressive effects are mediated by repressing Slug expression | [1] |
|  | Pancreatic cancer | Mcl-1 | Triptolide mediated miR-204 increase causes pancreatic cancer cell death via loss of Mcl-1 | [2] |
|  | gastric cancer | SIRT1 | down-regulation of miR-204 promotes gastric cancer cell invasion by activating the SIRT1-LKB1 pathway | [3] |
|  | gastric cancers | Bcl-2 | Ectopic expression of miR-204 inhibited colony forming ability, migration and tumor engraftment of GC cells by targeting Bcl-2 messenger RNA | [4] |
|  | endometrial cancer | FOXC1 | miR-204 inhibited  migration, invasion and extracellular matrix-adhesion in HEC1A endometrial cancer cells by targeting FOXC1 | [5] |
| miR-138 | non-small cell lung cancer | PDK1 | miR-138 could inhibit cell proliferation by targeting PDK1 in NSCLC cells | [6] |
|  | Colorectal cancer | TWIST2 | miR-138 regulated CRC metastasis by targeting TWIST2 | [7] |
|  | ovarian cancer | SOX4  HIF-1α | miR-138 inhibited ovarian cancer cell invasion and metastasis by targeting SOX4 and HIF-1α | [8] |
|  | non-small cell lung cancer | EZH2 | The EZH2 oncogene, which is often overexpressed in various human cancers and acts as an important regulator of cell growth and  tumor invasion, was identified as a novel target of miR-138 | [9] |
|  | nasopharyngeal carcinoma | CCND1 | The ectopic expression of miR-138dramatically suppressed cell proliferation and colony formation in vitro and inhibited tumorigenesis in vivo by targeting CCND1 | [10] |
| miR-146a | breast cancer | CXCR4 | TRAIL-induced miR-146a expression suppresses CXCR4-mediated human breast cancer migration, and provide further insight into the non-apoptotic function of TRAIL in the prevention of metastasis as a therapy for breast cancer | [11] |
|  | gastric cancer | UHRF1 | miR-146a/UHRF1 axis provides insight into the GC metastasis process, and targeting this novel axis represents a therapeutic approach to blocking GC metastasis | [12] |
| miR-195-star | non-small cell lung  cancer | MYB | Overexpression of MYB in NSCLC cells using an ectopic expression vector restored the decreased cell proliferation, migration and invasion effects induced by miR-195 | [13] |
|  | esophageal  squamous  cell carcinoma | Cdc42 | Ectopic expression of miR-195 in ESCC cells significantly downregulated Cdc42 by directly binding its 3' untranslated regions, and induced G1 cell cycle arrest, leading to a significant decrease in cell growth, migration, and invasion in vitro | [14] |
|  | human embryonic   stem cells | ARL2 | miR-195 regulates cell apoptosis in a context-dependent manner through directly targeting ARL2 | [15] |
|  | hepatocellular  carcinoma | IKKα  TAB3 | miR-195 suppresses cancer cell proliferation and migration in vitro and reduces tumorigenicity and metastasis in vivo by way of the direct targeting of IKKα and TAB3 | [16] |
| miR-328-star | gastrointestinal  cancer | CD44 | macrophages in the tumor microenvironment may cause increased CD44 expression through miR-328 suppression, resulting in tumor progression by enhancing ROS defense | [17] |
| miR-196b-star | chronic myeloid  leukemia | BCR-ABL1 HOXA9 | miR-196b acts to reduce BCR-ABL1 and HOXA9 protein levels, decrease cell proliferation rate and retard the cell cycle | [18] |
|  | cervical cancer | HOXB7 | miR-196b/HOXB7/VEGF pathway plays an important role incervical cancer progression; hence targeting this pathway could be a promising therapeutic strategy for the future management of this disease | [19] |
| miR-16-1-star | Osteosarcoma | CCND1 | miR-16-1 downregulate CCND1 and induce apoptosis and cell cycle arrest in osteosarcoma | [20] |
| miR-383 | testicular embryonal carcinoma | PNUTS | miR-383 impairs the phosphorylation of H2AX by targeting PNUTS and inducing cell cycle arrest independently, as well as sensitizing NT-2 cells to cisplatin | [21] |
|  | glioma cell | IGF1R | IGF1R expression is critical for miR-383 downregulation-induced cell invasion | [22] |
|  | medulloblastoma | PRDX3 | miR-383 acts as a regulator controlling cell growth of MB, at least in part, through targeting PRDX3 | [23] |
| miR-129-5p | Cervical Cancer | SP1 | Exogenous miR-129-5p inhibits cell proliferation in Hela cells, promotes apoptosis and blocks cell cycle progression in Hela cells | [24] |
|  | hepatocellular  carcinoma | VCP | miR-129-5p could inhibit the degradation of IκBα and increase the apoptosis and reduce the migration of HCC cells by suppressing the expression of VCP | [25] |
| miR-574-5p | colorectal cancer | Quaking | miR-574-5p  regulate Qki isoforms (Qki6/7 in particular) post-transcriptionally and caused altered expression in β-catenin and p27(Kip1) , increased proliferation, migration and invasion and decreased differentiation and cell cycle exit | [26] |
|  | multiple human cancer cell lines | CerS1-2 | Interference with HDAC1 and miR-574-5p reconstituted CerS1-2 expression and C18-ceramide generation in multiple human cancer cell lines, which subsequently inhibited proliferation and anchorage-independent growth | [27] |
| miR-296-3p | Mammalian pancreatic α and β cells | IGF1Rβ TNFα | raised the propensity to apoptosis of transfected and cytokine-treated αTC1-6 cells with respect to αTC1-6 cells, treated with cytokines after transfection with scramble molecules | [28] |
| miR-135a | Renal cell carcinoma | c-MYC | Restoration of mature miR-135a significantly inhibited cancer cell proliferation and induced G0/G1 arrest in the RCC cell lines caki2 and A498 | [29] |
| miR-125a | hepatocellular carcinoma | MMP11 VEGF-A | MiR-125a inhibits the proliferation and metastasis of HCC by targeting MMP11 and VEGF-A | [30] |
|  | human breast cancer | ERBB2 ERBB3 | miR-125a overexpression produced marginal influences on the growth and migration of these non-transformed human mammary epithelial cells | [31] |
| miR-24-2-star | breast cancer | PKCα | overexpression of miR-24-2* results in a dampening of cell survival through the targeted suppression of PKCα | [32] |
|  | breast cancer | BCL-2 | mir-24-2 is capable of inducing apoptosis by modulating different apoptotic pathways and targeting BCL-2, an antiapoptotic gene | [33] |
| miR-23a | thymic Lymphoma | Fas | miR-23a induced cell apoptosis by targeting Fas in Radiation-Induced thymic Lymphoma | [33] |
| miR-27a | esophageal squamous cell carcinoma | KRAS | miR-27a exerts its tumor suppressor function through inhibition of the KRAS-related ERK pathways | [34] |
| miR-147 | colon cancer | P27  Cyclin D1 | miR-147 was identified to: 1. cause MET primarily by increasing the expression of CDH1 and decreasing that of ZEB1; 2. inhibit the invasion and motility of cells; 3. cause G1 arrest by up-regulating p27 and down-regulating cyclin D1 | [35] |
| miR-132 | hepatocellular carcinoma | Akt | proliferation and colony formation of HCC cells were found to be suppressed by miR-132-mediated inhibition of the Akt-signaling pathway in miR132 transfected cells | [36] |
| miR-615-5p | heptocellular  carcinoma | IGF-II | Forced miR-615-5p expression in HCC cell lines led to significant decrease in cell growth and migration. | [37] |
| miR-760 | colorectal cancer cells | p53, p21 | miR-186, miR-216b, miR-337-3p, and miR-760 cooperatively promote cellular senescence through the p53-p21(Cip1/WAF1) pathway by CKII downregulation-mediated ROS production in HCT116 cells  miR-760 can potentially serve as promising non-invasive biomarkers for the early detection of CRC | [38][39] |
| miR-877 | hepatocellular carcinoma | - | miRNA expression associated with paclitaxel-induced apoptosis in hepatocellular carcinoma cells. | [40] |

**References:**

1. Qiu, Y.H., Wei, Y.P., Shen, N.J., Wang, Z.C., Kan, T., Yu, W.L., Yi, B. and Zhang, Y.J. (2013) miR-204 inhibits epithelial to mesenchymal transition by targeting slug in intrahepatic cholangiocarcinoma cells. *Cell Physiol Biochem*, **32**, 1331-41.

2. Chen, Z., Sangwan, V., Banerjee, S., Mackenzie, T., Dudeja, V., Li, X., Wang, H., Vickers, S.M. and Saluja, A.K. (2013) miR-204 mediated loss of Myeloid cell leukemia-1 results in pancreatic cancer cell death. *Mol Cancer*, **12**, 105.

3. Zhang, L., Wang, X. and Chen, P. (2013) MiR-204 down regulates SIRT1 and reverts SIRT1-induced epithelial-mesenchymal transition, anoikis resistance and invasion in gastric cancer cells. *BMC Cancer*, **13**, 290.

4. Sacconi, A., Biagioni, F., Canu, V., Mori, F., Di Benedetto, A., Lorenzon, L., Ercolani, C., Di Agostino, S., Cambria, A.M., Germoni, S., Grasso, G., Blandino, R., Panebianco, V., Ziparo, V., Federici, O., Muti, P., Strano, S., Carboni, F., Mottolese, M., Diodoro, M., Pescarmona, E., Garofalo, A. and Blandino, G. (2012) miR-204 targets Bcl-2 expression and enhances responsiveness of gastric cancer. *Cell Death Dis*, **3**, e423.

5. Chung, T.K., Lau, T.S., Cheung, T.H., Yim, S.F., Lo, K.W., Siu, N.S., Chan, L.K., Yu, M.Y., Kwong, J., Doran, G., Barroilhet, L.M., Ng, A.S., Wong, R.R., Wang, V.W., Mok, S.C., Smith, D.I., Berkowitz, R.S. and Wong, Y.F. (2012) Dysregulation of microRNA-204 mediates migration and invasion of endometrial cancer by regulating FOXC1. *Int J Cancer*, **130**, 1036-45.

6. Ye, X.W., Yu, H., Jin, Y.K., Jing, X.T., Xu, M., Wan, Z.F. and Zhang, X.Y. (2014) miR-138 inhibits proliferation by targeting 3-phosphoinositide-dependent protein kinase-1 in non-small cell lung cancer cells. *Clin Respir J*.

7. Long, L., Huang, G., Zhu, H., Guo, Y., Liu, Y. and Huo, J. (2013) Down-regulation of miR-138 promotes colorectal cancer metastasis via directly targeting TWIST2. *J Transl Med*, **11**, 275.

8. Yeh, Y.M., Chuang, C.M., Chao, K.C. and Wang, L.H. (2013) MicroRNA-138 suppresses ovarian cancer cell invasion and metastasis by targeting SOX4 and HIF-1alpha. *Int J Cancer*, **133**, 867-78.

9. Zhang, H., Zhang, H., Zhao, M., Lv, Z., Zhang, X., Qin, X., Wang, H., Wang, S., Su, J., Lv, X., Liu, H., Du, W., Zhou, W., Chen, X. and Fei, K. (2013) MiR-138 inhibits tumor growth through repression of EZH2 in non-small cell lung cancer. *Cell Physiol Biochem*, **31**, 56-65.

10. Liu, X., Lv, X.B., Wang, X.P., Sang, Y., Xu, S., Hu, K., Wu, M., Liang, Y., Liu, P., Tang, J., Lu, W.H., Feng, Q.S., Chen, L.Z., Qian, C.N., Bei, J.X., Kang, T. and Zeng, Y.X. (2012) MiR-138 suppressed nasopharyngeal carcinoma growth and tumorigenesis by targeting the CCND1 oncogene. *Cell Cycle*, **11**, 2495-506.

11. Wang, D., Liu, D., Gao, J., Liu, M., Liu, S., Jiang, M., Liu, Y. and Zheng, D. (2013) TRAIL-induced miR-146a expression suppresses CXCR4-mediated human breast cancer migration. *FEBS J*, **280**, 3340-53.

12. Zhou, L., Zhao, X., Han, Y., Lu, Y., Shang, Y., Liu, C., Li, T., Jin, Z., Fan, D. and Wu, K. (2013) Regulation of UHRF1 by miR-146a/b modulates gastric cancer invasion and metastasis. *FASEB J*, **27**, 4929-39.

13. Yongchun, Z., Linwei, T., Xicai, W., Lianhua, Y., Guangqiang, Z., Ming, Y., Guangjian, L., Yujie, L. and Yunchao, H. (2014) MicroRNA-195 inhibits non-small cell lung cancer cell proliferation, migration and invasion by targeting MYB. *Cancer Lett*.

14. Fu, M.G., Li, S., Yu, T.T., Qian, L.J., Cao, R.S., Zhu, H., Xiao, B., Jiao, C.H., Tang, N.N., Ma, J.J., Hua, J., Zhang, W.F., Zhang, H.J. and Shi, R.H. (2013) Differential expression of miR-195 in esophageal squamous cell carcinoma and miR-195 expression inhibits tumor cell proliferation and invasion by targeting of Cdc42. *FEBS Lett*, **587**, 3471-9.

15. Zhou, Y., Jiang, H., Gu, J., Tang, Y., Shen, N. and Jin, Y. (2013) MicroRNA-195 targets ADP-ribosylation factor-like protein 2 to induce apoptosis in human embryonic stem cell-derived neural progenitor cells. *Cell Death Dis*, **4**, e695.

16. Ding, J., Huang, S., Wang, Y., Tian, Q., Zha, R., Shi, H., Wang, Q., Ge, C., Chen, T., Zhao, Y., Liang, L., Li, J. and He, X. (2013) Genome-wide screening reveals that miR-195 targets the TNF-alpha/NF-kappaB pathway by down-regulating IkappaB kinase alpha and TAB3 in hepatocellular carcinoma. *Hepatology*, **58**, 654-66.

17. Ishimoto, T., Sugihara, H., Watanabe, M., Sawayama, H., Iwatsuki, M., Baba, Y., Okabe, H., Hidaka, K., Yokoyama, N., Miyake, K., Yoshikawa, M., Nagano, O., Komohara, Y., Takeya, M., Saya, H. and Baba, H. (2013) Macrophage-derived reactive oxygen species suppress miR-328 targeting CD44 in cancer cells and promote redox adaptation. *Carcinogenesis*.

18. Liu, Y., Zheng, W., Song, Y., Ma, W. and Yin, H. (2013) Low expression of miR-196b enhances the expression of BCR-ABL1 and HOXA9 oncogenes in chronic myeloid leukemogenesis. *PLoS One*, **8**, e68442.

19. How, C., Hui, A.B., Alajez, N.M., Shi, W., Boutros, P.C., Clarke, B.A., Yan, R., Pintilie, M., Fyles, A., Hedley, D.W., Hill, R.P., Milosevic, M. and Liu, F.F. (2013) MicroRNA-196b regulates the homeobox B7-vascular endothelial growth factor axis in cervical cancer. *PLoS One*, **8**, e67846.

20. Cai, C.K., Zhao, G.Y., Tian, L.Y., Liu, L., Yan, K., Ma, Y.L., Ji, Z.W., Li, X.X., Han, K., Gao, J., Qiu, X.C., Fan, Q.Y., Yang, T.T. and Ma, B.A. (2012) miR-15a and miR-16-1 downregulate CCND1 and induce apoptosis and cell cycle arrest in osteosarcoma. *Oncol Rep*, **28**, 1764-70.

21. Huang, H., Tian, H., Duan, Z., Cao, Y., Zhang, X.S. and Sun, F. (2014) microRNA-383 impairs phosphorylation of H2AX by targeting PNUTS and inducing cell cycle arrest in testicular embryonal carcinoma cells. *Cell Signal*, **26**, 903-911.

22. He, Z., Cen, D., Luo, X., Li, D., Li, P., Liang, L. and Meng, Z. (2013) Downregulation of miR-383 promotes glioma cell invasion by targeting insulin-like growth factor 1 receptor. *Med Oncol*, **30**, 557.

23. Li, K.K., Pang, J.C., Lau, K.M., Zhou, L., Mao, Y., Wang, Y., Poon, W.S. and Ng, H.K. (2013) MiR-383 is downregulated in medulloblastoma and targets peroxiredoxin 3 (PRDX3). *Brain Pathol*, **23**, 413-25.

24. Zhang, J., Li, S., Yan, Q., Chen, X., Yang, Y., Liu, X. and Wan, X. (2013) Interferon-beta Induced microRNA-129-5p Down-Regulates HPV-18 E6 and E7 Viral Gene Expression by Targeting SP1 in Cervical Cancer Cells. *PLoS One*, **8**, e81366.

25. Liu, Y., Hei, Y., Shu, Q., Dong, J., Gao, Y., Fu, H., Zheng, X. and Yang, G. (2012) VCP/p97, down-regulated by microRNA-129-5p, could regulate the progression of hepatocellular carcinoma. *PLoS One*, **7**, e35800.

26. Ji, S., Ye, G., Zhang, J., Wang, L., Wang, T., Wang, Z., Zhang, T., Wang, G., Guo, Z., Luo, Y., Cai, J. and Yang, J.Y. (2013) miR-574-5p negatively regulates Qki6/7 to impact beta-catenin/Wnt signalling and the development of colorectal cancer. *Gut*, **62**, 716-26.

27. Meyers-Needham, M., Ponnusamy, S., Gencer, S., Jiang, W., Thomas, R.J., Senkal, C.E. and Ogretmen, B. (2012) Concerted functions of HDAC1 and microRNA-574-5p repress alternatively spliced ceramide synthase 1 expression in human cancer cells. *EMBO Mol Med*, **4**, 78-92.

28. Barbagallo, D., Piro, S., Condorelli, A.G., Mascali, L.G., Urbano, F., Parrinello, N., Monello, A., Statello, L., Ragusa, M., Rabuazzo, A.M., Di Pietro, C., Purrello, F. and Purrello, M. (2013) miR-296-3p, miR-298-5p and their downstream networks are causally involved in the higher resistance of mammalian pancreatic alpha cells to cytokine-induced apoptosis as compared to beta cells. *BMC Genomics*, **14**, 62.

29. Yamada, Y., Hidaka, H., Seki, N., Yoshino, H., Yamasaki, T., Itesako, T., Nakagawa, M. and Enokida, H. (2013) Tumor-suppressive microRNA-135a inhibits cancer cell proliferation by targeting the c-MYC oncogene in renal cell carcinoma. *Cancer Sci*, **104**, 304-12.

30. Bi, Q., Tang, S., Xia, L., Du, R., Fan, R., Gao, L., Jin, J., Liang, S., Chen, Z., Xu, G., Nie, Y., Wu, K., Liu, J., Shi, Y., Ding, J. and Fan, D. (2012) Ectopic expression of MiR-125a inhibits the proliferation and metastasis of hepatocellular carcinoma by targeting MMP11 and VEGF. *PLoS One*, **7**, e40169.

31. Scott, G.K., Goga, A., Bhaumik, D., Berger, C.E., Sullivan, C.S. and Benz, C.C. (2007) Coordinate suppression of ERBB2 and ERBB3 by enforced expression of micro-RNA miR-125a or miR-125b. *J Biol Chem*, **282**, 1479-86.

32. Martin, E.C., Elliott, S., Rhodes, L.V., Antoon, J.W., Fewell, C., Zhu, Y., Driver, J.L., Jodari-Karimi, M., Taylor, C.W., Flemington, E.K., Beckman, B.S., Collins-Burow, B.M. and Burow, M.E. (2014) Preferential star strand biogenesis of pre-miR-24-2 targets PKC-alpha and suppresses cell survival in MCF-7 breast cancer cells. *Mol Carcinog*, **53**, 38-48.

33. Srivastava, N., Manvati, S., Srivastava, A., Pal, R., Kalaiarasan, P., Chattopadhyay, S., Gochhait, S., Dua, R. and Bamezai, R.N. (2011) miR-24-2 controls H2AFX expression regardless of gene copy number alteration and induces apoptosis by targeting antiapoptotic gene BCL-2: a potential for therapeutic intervention. *Breast Cancer Res*, **13**, R39.

34. Zhu, L., Wang, Z., Fan, Q., Wang, R. and Sun, Y. (2014) microRNA-27a functions as a tumor suppressor in esophageal squamous cell carcinoma by targeting KRAS. *Oncol Rep*, **31**, 280-6.

35. Lee, C.G., McCarthy, S., Gruidl, M., Timme, C. and Yeatman, T.J. (2014) MicroRNA-147 Induces a Mesenchymal-To-Epithelial Transition (MET) and Reverses EGFR Inhibitor Resistance. *PLoS One*, **9**, e84597.

36. Wei, X., Tan, C., Tang, C., Ren, G., Xiang, T., Qiu, Z., Liu, R. and Wu, Z. (2013) Epigenetic repression of miR-132 expression by the hepatitis B virus x protein in hepatitis B virus-related hepatocellular carcinoma. *Cell Signal*, **25**, 1037-43.

37. El Tayebi, H.M., Hosny, K.A., Esmat, G., Breuhahn, K. and Abdelaziz, A.I. (2012) miR-615-5p is restrictedly expressed in cirrhotic and cancerous liver tissues and its overexpression alleviates the tumorigenic effects in hepatocellular carcinoma. *FEBS Lett*, **586**, 3309-16.

38. Kim, S.Y., Lee, Y.H. and Bae, Y.S. (2012) MiR-186, miR-216b, miR-337-3p, and miR-760 cooperatively induce cellular senescence by targeting alpha subunit of protein kinase CKII in human colorectal cancer cells. *Biochem Biophys Res Commun*, **429**, 173-9.

39. Wang, Q., Huang, Z., Ni, S., Xiao, X., Xu, Q., Wang, L., Huang, D., Tan, C., Sheng, W. and Du, X. (2012) Plasma miR-601 and miR-760 are novel biomarkers for the early detection of colorectal cancer. *PLoS One*, **7**, e44398.

40. Yan, H., Wang, S., Yu, H., Zhu, J. and Chen, C. (2013) Molecular pathways and functional analysis of miRNA expression associated with paclitaxel-induced apoptosis in hepatocellular carcinoma cells. *Pharmacology*, **92**, 167-74.
